# The Alarming Situation of Highly Pathogenic Avian Influenza Viruses in 2019–2023

**DOI:** 10.1055/s-0044-1788039

**Published:** 2024-06-28

**Authors:** Zhiwei Zhang, Zhao Lei

**Affiliations:** 1 State Key Laboratory of Molecular Vaccinology and Molecular Diagnostics, School of Public Health, Xiamen University, Xiamen, Fujian Province, People's Republic of China; 2 Department of Industrial & Systems Engineering, The Hong Kong Polytechnic University, Kowloon, Hong Kong

**Keywords:** highly pathogenic avian influenza viruses, epidemiology, transmission, prevention and control

## Abstract

Avian influenza viruses (AIVs) have the potential to cause severe illness in wild birds, domestic poultry, and humans. The ongoing circulation of highly pathogenic avian influenza viruses (HPAIVs) has presented significant challenges to global poultry industry and public health in recent years. This study aimed to elucidate the circulation of HPAIVs during 2019 to 2023. Specifically, we assess the alarming global spread and continuous evolution of HPAIVs. Moreover, we discuss their transmission and prevention strategies to provide valuable references for future prevention and control measures against AIVs.

## Introduction

Avian influenza viruses (AIVs), commonly known as bird flu, were described as influenza type A viruses within the genus *Alphainfluenzavirus* of the family Orthomyxoviridae. 1 2 They have the potential to cause severe illness in wild birds, domestic poultry, and humans. Over recent years, AIVs have presented significant challenges to global public health systems due to their extensive circulation and notable mortality rates. 3 Based on their pathogenicity to chickens ( *Gallus gallus domesticus* ), AIVs are classified into highly pathogenic AIVs (HPAIVs) and low pathogenic AIVs (LPAIVs). 4 HPAIVs have been a longstanding concern due to their capacity to induce severe disease and fatalities in various bird species, including ducks and geese, 5 6 7 8 9 and their ability to spill over into other mammalian species, such as swine, seals, and humans, 7 10 11 12 13 resulting in significant impacts on poultry populations and posing a considerable threat to human health. HPAIVs have been responsible for the loss of approximately 500 million domestic birds and hundreds to thousands of human infections, with a high case fatality rate of 30 to 50%. Given the substantial economic losses to the poultry industry and the potential for a pandemic, continuous monitoring of HPAIVs is crucial.

AIVs possess segmented, single-stranded, negative-sense RNA genomes, with a length of approximately 13.5 kb. 14 The AIV genome consists of 8 segments: polymerase basic protein 2 (PB2), PB1, polymerase acidic (PA), hemagglutinin (HA), neuraminidase (NA), nucleoprotein (NP), matrix (M), and nonstructural (NS), which encode at least 10 structural and 9 regulatory proteins. 15 16 17 18 19 The viral genome undergoes continuous antigenic shift and antigenic drift during host infections, leading to the emergence of novel viral strains capable of evading existing host immunity. 20 AIVs are further categorized into multiple subtypes based on the antigenicity and genetic variation of the genes encoding the two surface glycoproteins: HA and NA. Presently, there are 16 different HA (H1–16) and 9 different NA (N1–9) subtypes detected in wild bird populations. 21 22 23 According to the World Health Organization (WHO), the HA genes of different lineages are assigned unified nomenclature to track the evolution of phylogenetically distinct Clades 0–9, 24 25 with Clade 2 further divided into five subclades, 2.1–2.5. 26 These subclades are further subdivided into 2.1.1–2.3.4, respectively. HPAIVs from Clade 2.3.4 have been further categorized into four subclades, 2.3.4.1–2.3.4.4. 27 28 29 Due to the global evolution and spread of Clade 2.3.4.4, it has evolved into eight subclades, designated as 2.3.4.4a–2.3.4.4h. 30

The majority of current circulating H5 HPAIVs belong to Clade 2. For instance, Clade 2.2 H5N1 HPAIVs of the Guangdong H5 lineage spread from China to Siberia and subsequently to several countries in Asia, Europe, the Middle East, and Africa during the epidemiologic year 2005 to 2006. 31 32 33 Since first reported in migratory birds in eastern China in 2013, Clade 2.3.4.4 H5 viruses have caused numerous outbreaks worldwide in both domestic and wild birds. Clade 2.3.4.4a mainly refers to the H5N6 viruses that emerged in China in 2014, while Clades 2.3.4.4b and 2.3.4.4c include the H5N8 viruses associated with intercontinental outbreaks in Asia, Europe, the United States., Canada, and Africa between 2014 and 2018. 34 These genetically distinct subgroups emerged and spread along different flyways into Europe, North America, and East Asia, respectively. Clade 2.3.4.4c comprises the H5N8 AIVs responsible for the avian flu outbreak in South Korea in 2014 35 and subsequently detected in America and Europe, along with the reassortment of H5N2 viruses, which caused poultry outbreaks in wild waterfowl, backyard, and commercial poultry along the Pacific flyway. 36 Clades 2.3.4.4d and 2.3.4.4h consist of H5N6 viruses isolated from samples in China. Clade 2.3.4.4e mainly comprises viruses from South Korea and Japan during 2016 to 2017, while Clades 2.3.4.4f and 2.3.4.4 g primarily refer to H5N1 and H5N6 viruses found in China and neighboring southwestern countries, respectively. 29 37 Moreover, within the current HPAIVs H5 2.3.4.4 clade, some defined subclades have undergone substantial reassortment with LPAIVs of wild birds. 38 Additionally, among the various subtypes, sporadic human infections have been reported with H5, H6, H7, H9, and H10 subtypes. 39 40 The predominant HPAIVs currently identified belong to H5 and H7 subtypes, including H5N1, H5N6, H5N8, H7N3, H7N7, and H7N9. 41 42

As pathogens affecting a wide range of species, HPAIVs are perpetuated and circulated not only among avian species but also among other nonavian animals and humans. Wild aquatic birds, including Anseriformes (e.g., geese, ducks) and Charadriiformes (e.g., gulls, shorebirds), are considered the primary reservoirs of these pathogens. 33 Additionally, HPAIVs have been isolated in domestic avian hosts, particularly Galliformes (e.g., chicken, turkey), causing sporadic infections and sustained transmission in domestic poultry. 43 44 These viruses can devastate poultry populations, resulting in significant economic losses in the agriculture sector. While wild birds and poultry are the primary hosts of HPAIVs, some subtypes can cross species barriers to infect mammals, often with fatal outcomes. Thus, sporadic infections have been reported in mammals, including wild or feral animals (e.g., foxes, seals), stray or domestic animals (e.g., cats, dogs), and zoo animals (e.g., tigers, leopards). 45 46 47 48 Some mammals are suggested to act as mixing vessels for influenza viruses, potentially leading to the emergence of new viruses with improved infectivity for animals and humans. Human infections with HPAIVs have been reported in numerous countries, particularly in the last 5 years, typically following unprotected exposures to infected poultry or virus-contaminated environments. Although rare, sporadic human infections have occurred. Consequently, as a potential pandemic threat, HPAIVs have garnered significant attention.

HPAIVs can induce a spectrum of symptoms in infected individuals, ranging from none or mild illness to severe disease and fatalities. Reported symptoms in humans include mild upper respiratory tract symptoms, lower respiratory tract disease, severe pneumonia with respiratory failure, encephalitis, gastrointestinal and neurological symptoms, multi-organ failure, and/or death. Since 2016, sporadic human infections with HPAIVs have been reported globally each year. For instance, one case of asymptomatic infection was reported in Vietnam in 2011, and another asymptomatic case occurred in the United Kingdom in late 2021. However, the case fatality rate for some H5 and H7 subtype HPAIV infections among humans has been shown to be higher than that of seasonal influenza infections. For example, among human clinical cases of H5N1 HPAIV reported to date, the case fatality rate exceeds 52%. 49 Therefore, understanding these viruses is crucial for developing strategies for prevention, treatment, and outbreak containment.

To mitigate the impact of highly pathogenic avian influenza (HPAI) on poultry industries and reduce the risk of human infections, monitoring the spread of avian influenza is essential. Surveillance programs and related organizations, such as the Global Influenza Surveillance and Response System (GISRS) Collaborating Centers for Reference and Research on Influenza developed by the WHO, the World Animal Health Information System (WAHIS) provided by the World Organization for Animal Health (WOAH), the Food and Agriculture Organization of the United Nations (FAO), the Animal and Plant Health Inspection Service (APHIS) by the U.S. Department of Agriculture (USDA), the Public Health Agency of Canada, China's Centers for Disease Control, and the passive surveillance system for testing wild birds found dead or sick for avian influenza in European Union member states since 2005, 50 serve as early warning systems at both global and regional levels. Recent detections of HPAI highlight the ongoing risk these viruses pose to animal and human health, emphasizing the need for continuous vigilance and research to understand their evolving nature and modes of transmission. Efforts to control avian influenza include promoting transparency, enhancing understanding of HPAIVs, and implementing crucial biosecurity measures to protect the poultry industry and prevent potential human infections. 4 Given the alarming global spread and continuous evolution of HPAIVs and their impact on the poultry industry and public health, there is an urgent need to review the latest science and evidence on HPAI and control strategies.

## Outbreaks and Epidemiology of HPAIVs

Since their first identification in 1996, HPAIVs have spread across multiple continents, resulting in a notable rise in the frequency of avian influenza outbreaks in poultry, other avian species, and mammals in recent years. Consequently, HPAIVs have prompted global concerns due to their high pathogenicity in poultry and elevated fatality rates in humans, particularly during the past 5 years from 2019 to 2023.

### The Global Distribution of HPAIVs

**Fig. 1:**
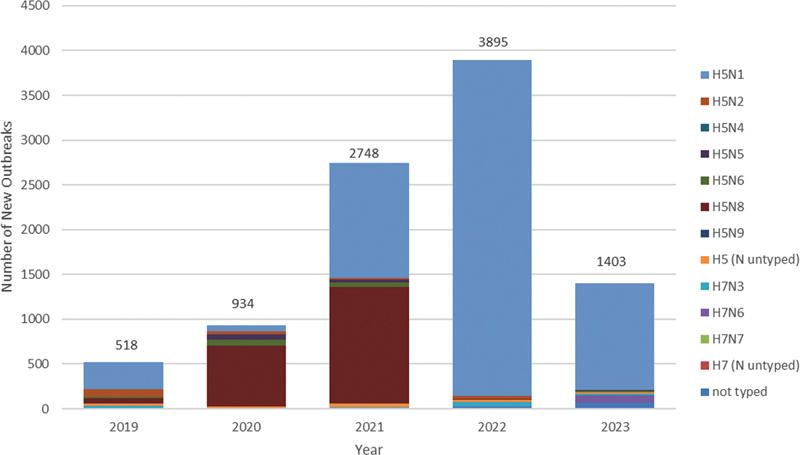
The avian influenza outbreaks caused by various HPAIVs occurred in 2019 to 2023. HPAIVs, highly pathogenic avian influenza viruses.

According to data from the WAHIS ( https://wahis.woah.org/#/dashboards/qd-dashboard ), a total of 9,498 avian influenza outbreaks caused by various HPAIVs have occurred from 2019 to 2023 ( Fig. 1 ). These outbreaks have resulted in nearly 60 million cases worldwide, with approximately 24.8 million poultry lost during this period. A wide range of domestic birds and animals, including birds, Anseriformes, Galliformes, have been affected in the past 5 years ( Supplementary Table S1 [online only]).

**Fig. 2:**
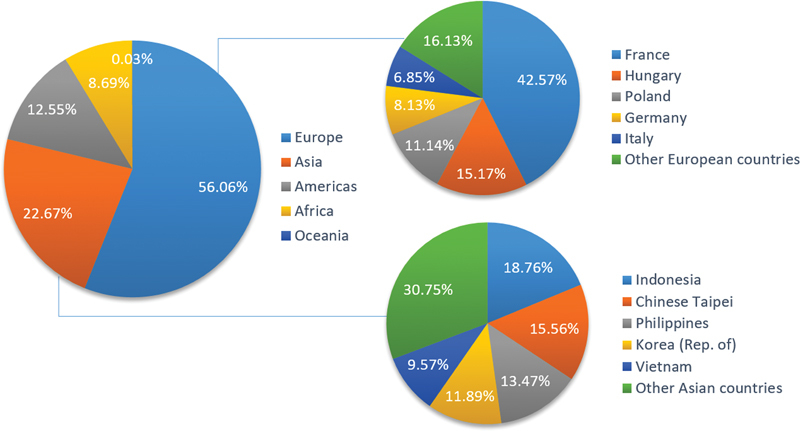
Global distribution of the HPAIVs outbreaks in 2019 to 2023. HPAIVs, highly pathogenic avian influenza viruses.

During the period from 2019 to 2023, a total of 80 countries or regions across five continents—Africa, the Americas, Asia, Europe, and Oceania—reported HPAI outbreaks in both wild birds and poultry to WOAH ( https://www.who.int/news/item/12-07-2023-ongoing-avian-influenza-outbreaks-in-animals-pose-risk-to-humans ). Among these continents, approximately 56.1% of outbreaks occurred in Europe during the recent 5-year period ( Fig. 2 ). France recorded the highest number of HPAI outbreaks, primarily caused by H5N1 and H5N8 viruses between 2020 and 2023. Other European countries, including Hungary, Poland, and Germany, also reported relatively high numbers of outbreaks caused by H5N1, H5N2, H5N5, and H5N8 viruses. Apart from Europe, avian influenza outbreaks caused by HPAIVs were predominantly reported in various countries across other continents, including the United States of America in the Americas, Nigeria in Africa, and Indonesia in Asia.

### Damage to Animals

The global spread and significant increase in HPAI outbreaks have affected both domestic and wild animals. Over 294 million domestic poultry have been lost due to death or culling in affected areas worldwide. Numerous mass death events have been reported in both terrestrial and aquatic animals, including birds (Anseriformes, Galliformes), cattle, Equidae, sheep, goats, and swine, with at least 13 species affected. It is likely that there are more animals affected by outbreaks that have yet to be detected or reported. In 2021, two European countries—the United Kingdom and Bulgaria—reported outbreaks in wild animals, primarily in Common pheasants and Phasianidae, resulting in the deaths of over 800 animals. This shift in the epidemiology and ecology of HPAIVs has heightened global concern as the spread of these viruses has led to a concerning increase in cases among birds and mammals, posing threats to public health, animal health, food security, and biodiversity. 51

### Assessing the Risk to Humans

Sporadic human infection cases by the HPAI influenza virus were reported during 2019 to 2023 but remained very rare. According to information available on WOAH, there were 90 human cases confirmed by national authorities or WHO during the past 5 years, including 21 deaths, with a case fatality proportion of approximately 23%. It has been shown that HPAI infections in humans could cause severe disease with a high mortality rate.

Since early 2019, HPAI strains, notably H5N6, H5N1, and H5N8 subtypes, caused significant human infection cases worldwide. The majority of these human infection cases occurred during 2021 to 2023 in Asia and European countries, including China, Cambodia, the United Kingdom., the Russian Federation, and others. The human cases detected so far are mostly linked to close contact with infected poultry or contaminated environments. A comparison of HA sequences from human- and avian-derived HPAIVs indicated high homology within the same subtype of viruses. 52

Based on the information available, the virus does not appear to transmit easily from one person to another. However, studies monitoring the epidemiology and evolution of the HPAIVs, identifying signals of any changes of the HPAIVs that could be more threatening to infect humans, are of significant importance for risk assessment and disease control.

## Pathogenesis and Transmission of HPAIVs

HPAIVs have continually caused worldwide outbreaks in wild birds, poultry, and mammals, with some spillover to humans. Most notably, HPAIVs are primarily composed of H5 and H7 viruses, infections with which have resulted in significant mortality within susceptible poultry species and have become a major concern for public health as well.

### HPAI H5N1 Viruses

Since first emerging in domestic waterfowl in southern China in 1996, the HPAI H5N1 A/goose/Guangdong/1/1996 lineage has spread widely throughout Asia, followed by Africa, Europe, and the Middle East, leading to several large poultry outbreaks and sporadic human infections. From 2019 to 2023, 6,590 avian influenza outbreaks caused by HPAI H5N1 viruses were reported in several European, American, African, and Asian countries, including France, the United States, Hungary, Nigeria, Indonesia, etc. Migratory birds have been implicated in the dissemination of HPAI H5N1 viruses in Asia and from Asia to Europe. 53 54 Additionally, according to the information reported in the WAHIS ( https://wahis.woah.org ), the number of poultry lost in the outbreaks that occurred during these 5 years has been up to 218 million worldwide. Moreover, the first human infections with HPAI A(H5N1) virus were identified in Hong Kong, China in 1997, followed by several years without wide detection until it re-emerged across Asia in 2003. As of December 2023, 882 human cases of H5N1 virus infection and 461 deaths had been reported from 23 countries since 2003, with approximately 52.3% case fatality proportion, according to WHO, 49 including 14 cases and 6 deaths in 7 countries during 2019 to 2023.

The HPAI H5N1 virus circulating in wild birds and poultry worldwide has undergone genetic variations during the epidemic waves. During 2020, reassortment between poultry and wild bird viruses led to the emergence of a distinct HPAI clade 2.3.4.4b H5N1 viruses with an N1 NA from wild birds. 55 It was first identified in Europe during the fall of 2020 and spread across Europe and into Africa, the Middle East, and Asia, which has become the predominant subtype of HPAI H5 by the end of 2021. 56 In late 2021, the H5N1 HPAIV clade 2.3.4.4b was then isolated in poultry and a free-living gull in Canada; the high genetic identity with the wild bird H5N1 virus circulating in northwestern Europe in spring 2021 indicated the transatlantic spread via wild bird migration. 57 58 In February 2022, according to the USDA's APHIS, an HPAI H5N1 outbreak in turkeys was detected in a commercial poultry facility in the United States. 38 This wild-bird-adapted HPAIVs H5N1 have caused sporadic infections in wild birds and outbreaks in commercial and domestic poultry across multiple countries, with occasional spillover to mammals observed. 59 60 61

The detections of HPAI H5N1 virus in various mammals have been reported worldwide. In middle and late 2021, HPAI H5N1 virus clade 2.3.4.4b was detected in two wild red fox ( *Vulpes vulpes* ) cubs in the Netherlands and Estonia, respectively. 62 63 In 2022, Peru reported HPAI H5N1 virus infections in sea lions following the deaths of hundreds of sea lions. Later, HPAI H5N1 viruses of clade 2.3.4.4b also caused infections and some deaths in 10 seals in Maine, 64 among mink on a farm in northwest Spain, and so on, 12 in bears in Alaska, Nebraska, and Montana. 61 In 2023, HPAIV H5N1 was found to have infected a polar bear, as well as elephant and fur seals in the Antarctic, marking the first HPAI A(H5N1) virus infections in both polar regions. Other animals that have tested positive for HPAI H5 virus include otters, lynx, a polecat, and a badger in Europe, and raccoon dogs and foxes in Japan. 65

Sporadic infections of HPAI H5N1 virus in humans have also been reported globally. In January 2022, an asymptomatic 80-year-old man who raised ducks in England was reported to have HPAI H5N1 clade 2.3.4.4b virus infection. 66 The second human case associated with this specific group of H5 viruses was reported in the United States, involving direct exposure to poultry and participation in the culling of poultry with presumptive H5N1 bird flu. In late 2022, Spain reported two human cases of HPAI H5N1 in asymptomatic poultry workers from the same poultry farm with a confirmed outbreak of H5N1. China also reported a human case of HPAI H5N1 virus infection in an adult who developed critical illness and died after poultry exposure. Vietnam reported a case of human infection with HPAI A(H5) virus in a child who became critically ill following exposure to infected backyard poultry. In early 2023, Ecuador reported its first human infection with HPAI A(H5) in a child who became critically ill following exposure to infected backyard poultry. The Cambodia Ministry of Health reported two human infections with HPAI H5N1 virus, including one fatal case. Notably, in contrast to the HPAI H5N1 clade 2.3.4.4b virus circulating in wild birds and poultry in the United States and other countries, the viruses in Cambodia belong to clade 2.3.2.1c. 38

### HPAI H5N6 Viruses

H5N6 was first documented in Laos during 2013, subsequently spreading to Vietnam and China, as well as other countries via western and eastern routes. 27 67 Since 2013, HPAI subclade 2.3.4.4 H5N6 virus has been reported in wild birds and poultry in Asia, crossing geographical barriers to emerge in South Korea, Japan, and Europe as well. Phylogenetic analysis has shown that clade 2.3.4.4 H5N6 HPAIVs have continuously reassorted with other subtypes such as H5, H6, and H7N9/H9N2. 68

During 2019 to 2023, 145 outbreaks led by HPAI H5N6 have been documented by WOAH, resulting in more than 100,000 cases with more than half deaths in domestic birds, leading to the death or culling of approximately 1.4 million birds. The rise in the frequency of HPAI H5N6 virus infections, especially in 2020 and 2021, has raised serious concerns and posed an alarming threat to public health. 69 The first detection of HPAIV H5N6 (clade 2.3.4.4b) in Africa was reported in June 2019 in a duck from a live bird market in Nigeria, with the genome closely related to the H5N6 viruses detected in Europe during 2017 to 2018. 70 Full-length genome analysis of swab samples collected from live poultry markets in China in 2021 found that the 2.3.4.4b H5N6 viruses were novel reassortants, with the HA and M genes derived from 2.3.4.4b H5N8 viruses of wild-bird origin, whereas other internal genes exhibited several separate clusters. 69 Additionally, it has been documented that many other subtypes of AIVs from wild birds have donated the internal genes to H5N6 virus, leading to the diversification of this virus in both poultry and humans. 29 71 72 73 74 75 Transmissibility and receptor binding properties suggest that some H5N6 viruses acquired efficient in-contact transmission in ferrets, as well as binding affinity for human-like SAα2,6Gal-linked receptors, enabling attachment to human tracheal epithelial and alveolar cells. 76 Therefore, the high genetic diversity and virulence in mammals of H5N6 viruses pose an increasing threat to public health, necessitating close monitoring of continual adaptation in humans.

Most HPAI H5N6 virus human infection cases were reported in Asia, with almost all confirmed cases occurring in individuals with poultry exposure. By surveying epidemiological, clinical, and genetic data of human infection with H5N6 viruses, Zhu et al found that severe disease occurred in 93.8% of cases, with an estimated case fatality rate of 55.4%. 75 Since the first human infection case of HPAI H5N6 virus was documented in Sichuan Province, China, in 2014, 77 human cases have been continuously reported worldwide, caused by H5N6 HPAIVs belonging to clades 2.3.4.4a, 2.3.4.4b, 2.3.4.4d, 2.3.4.4 g, and 2.3.4.4h. 75 During 2019 to 2023, there have been 64 HPAI H5N6 virus human infections documented in China and one case reported in Laos, including 14 human deaths. 78 The clinical presentations of human infections with HPAI H5N6 virus often begin with fever, upper respiratory tract symptoms, and myalgia, and may rapidly progress to lower respiratory tract illness, resulting in pneumonia, multi-organ failure, acute respiratory distress syndrome, and often death. 28 In 2020, five human cases of HPAI H5N6 virus infection were reported in China. In 2021, Laos reported its first human infection with HPAI H5N6 virus in a child who had contact with infected poultry. Additionally, China reported 36 cases of human infection with HPAI H5N6 virus, including 18 deaths. In 2022, China reported 11 cases of HPAI H5N6 virus infection after poultry exposures, with severe or critical illness, including two deaths. Notably, most of these H5N6 HPAIV HA genes in humans during this period originated from subclade 2.3.4.4b.

### HPAI H5N8 Viruses

The HPAI H5N8 virus, belonging to H5 clade 2.3.4.4, was first detected in poultry in South Korea in 2014 79 and has since disseminated to both domestic and wild birds across Asia, Europe, and North America by mid-2015. During the 2020 to 2021 influenza season, novel H5N8 viruses repeatedly entered Europe, Russia, South Korea, and China, causing severe poultry and wild bird outbreaks in several European and Asian countries. 80 81 More than 2,000 HPAI H5N8 outbreaks have occurred during 2019 to 2023 worldwide, leading to nearly 9 million infection cases in wild and domestic birds and other animals, with over 4 million deaths. In early 2020, small H5N8 outbreaks were reported across the European poultry sector, followed by the detection of HPAIV H5N8 in poultry in the Republic of Iraq, Russian Federation, Kazakhstan, and the Netherlands since the middle of 2020. 55 From late 2020 to early 2021, HPAIVs H5N8 were isolated from swans, seals, and a fox in the United Kingdom, 82 Germany, 83 and Denmark. 84 In December 2020, the first human case of HPAI H5N8 virus infection was reported in an asymptomatic poultry worker in Russia, 85 suggesting that the H5N8 HPAI virus should be closely monitored for its potential threat to poultry production, food security, and public health.

The rapid global spread of H5N8 HPAIVs has been linked with wild bird migration. 83 86 87 88 89 The co-circulation of H5N8 and other subtypes of viruses in migratory birds has also accelerated the evolution of novel variants. It has been reported that the H5N8 virus reassorted with different viruses and formed several other subtypes of H5 viruses (e.g., the subtypes H5N1, H5N2, H5N3, H5N4, H5N5, and H5N6) in different countries and regions. 90 Since late 2019 and lasting until June 2020, outbreaks caused by novel HPAI clade 2.3.4.4b H5N8 viruses occurred in Central and Eastern European countries, which were generated by reassortment between H5N8 from sub-Saharan Africa and low-pathogenicity avian influenza (LPAI) viruses from Eurasia. 89 91 Two genetically distinct HPAI H5N8 viral lineages were detected to cause the outbreaks in Russia during August and September 2020, with one variant represented by viruses of clade 2.3.4.4b isolated in Europe in early 2020 and the other by a novel reassortant between clade 2.3.4.4b and Eurasian LPAI viruses circulating in wild birds, respectively. 55 92 The Danish H5N8 viruses found in late 2020 were shown to be genetically similar to contemporary European clade 2.3.4.4b H5N8 viruses, possibly new incursions introduced by migrating birds to overwintering sites in Europe. 84 Meanwhile, the Netherlands HPAI H5N8 detected in mute swans during the same period shared a common ancestor with clade 2.3.4.4b viruses detected in Egypt during 2018 to 2019 with a similar genetic composition, not directly related to H5N8 viruses from Europe detected in early 2020. 93 Moreover, in East Asia, HPAI H5N8 virus outbreaks 81 94 95 96 97 98 from late 2020 to early 2021 shared a recent common ancestor with 2.3.4.4b H5N8 HPAIVs that circulated in Europe in late 2020, whereas some HPAI H5N8 viruses detected in South Korea had a close genetic relationship with the European viruses from early 2020, 96 indicating the seasonal spread of HPAI virus lineages across the globe. 92 94 95

### HPAI H7N3 Viruses

Since its first isolation in 2012, 99 outbreaks of HPAI H7N3 viruses have been documented in various regions, including Mexico, British Columbia, Chile, Italy, and the United Kingdom. 100 Infections of HPAIVs H7N3 have been detected in both wild birds and domestic poultry. From 2019 to 2023, HPAI H7N3 viruses have mostly been isolated from samples in North America. Mexico and the United States. have experienced 108 H7N3 HPAI outbreaks, resulting in the death of more than 110,000 domestic birds. During the HPAI H7N3 outbreak in Mexico in June 2022, a total of 2.1 million birds from affected poultry production units and backyard farms were culled. 101 In March 2020, an outbreak of HPAI H7N3 occurred on U.S. turkey farms, further emphasizing the global spread and impact of these viruses. 102

Phylogenetic analyses have indicated that the Mexican H7N3 HPAIV originated from low-pathogenic North American wild bird AIV gene pools, followed by subsequent mutation to a highly pathogenic phenotype with complicated reassortment events. 103 Thereafter, the H7N3 HPAIVs evolved rapidly and diverged into multiple clusters. 104 105 Hesterberg et al studied the evolution of AIVs in wild birds, including H7N3, highlighting the complexity of virus evolution in a multi-host ecosystem and underlining the importance of monitoring wild bird populations for a comprehensive understanding of the diversity and evolution of AIVs. 31 Host adaptation of the Mexican H7N3 HPAI virus indicated high pathogenicity for chickens, whereas a loss of fitness was observed in mallards after circulation. 106 The emergence and selection of the HPAI H7N3 virus in a turkey farm in the Netherlands in 2020 provided direct evidence of LPAI to HPAI virus mutation, 107 potentially caused by the insertion or substitution of basic amino acids at the HA0 cleavage site as suggested in previous studies. 108 109 An epizootic of highly pathogenic H7N3 avian influenza in an ecologic reserve in Mexico underscores the importance of surveillance and biosecurity measures. 110 Understanding the pathobiological origins and evolutionary history of HPAIVs, including H7N3, is crucial for effective control and prevention strategies. 111 Measures such as culling infected flocks, disinfection of materials, and treatment with antiviral medications like oseltamivir have been used to control outbreaks of H7N3 avian influenza in various countries. 112

Although HPAI H7N3 virus infections have been reported in a small number of people with conjunctivitis since 2004 in Canada and other countries, 113 with some cases resulting in mild symptoms in humans, such as conjunctivitis and mild influenza-like illness, no human infection cases were reported during the recent 5 years. Genetic characterization as well as pathogenesis in both in vitro and in vivo models of AIV H7N3 isolated from spot-billed ducks in South Korea in early 2019 highlighted the potential for the viruses to mutate and infect humans, emphasizing the importance of routine surveillance. 114 Therefore, continued global surveillance of the H7N3 virus in wild birds and poultry populations is essential to understanding its ecology, evolution, and potential threat to humans.

### HPAI H7N7 Viruses

Since it was first recorded in 1927, the H7N7 subtype has been commonly isolated in surveillance studies of waterfowl in Europe and other parts of the world. 115 HPAIVs of the H7N7 subtype have been a cause of concern due to their virulence and potential to cause severe disease in poultry. During 2019 to 2023, Australia experienced three poultry outbreaks of HPAIVs H7N7 from July to December 2020, resulting in over 255,000 cases reported and the slaughter of more than 433,000 chickens. Scheibner et al 116 studied the virulence of three European H7 viruses in Pekin and Muscovy ducks. All the Muscovy ducklings inoculated with HPAIV H7N7 exhibited mild to severe clinical signs resulting in the death of 2 out of 10, while no impact of the infection route on virulence was observed in Pekin ducklings, indicating the variable virulence of HPAIV H7 in different duck species. Spruit et al 117 studied the N-glycolylneuraminic acid (NeuGc) binding of avian and equine H7 influenza A viruses, highlighting the extinct highly pathogenic equine H7N7 viruses that exclusively bind NeuGc. Vaccination has proven effective in reducing the transmission of H7N7 in chickens and preventing major outbreaks. 118 Engineered viral vaccine constructs have been developed to provide dual protection against H7N7 avian influenza and Newcastle disease in poultry. 119

The first documented HPAIV H7N7 human infection case occurred in the United States. in 1959, detected in the blood of a man clinically diagnosed with infectious hepatitis. 120 Since then, H7N7 HPAIV human infections have been reported in England, 121 the Netherlands, 122 Italy, 123 and other countries, associated with exposures during widespread poultry outbreaks. In these cases, mild upper respiratory tract symptoms, lower respiratory tract disease, severe pneumonia with respiratory failure, and multi-organ failure have been reported, including fatal cases. However, no HPAI H7N7 human infection cases were detected during 2019 to 2023. Research on previous H7N7 human cases has investigated the molecular basis of the viruses' pathogenicity to understand differences in virus pathogenicity. 124 Studies on contemporary North American H7 viruses have shown that they possess human receptor specificity, impacting virus transmissibility. 125 Additionally, the tropism and innate host responses of H7N7 and other influenza viruses have been studied to understand their pathogenesis in the human respiratory tract. 126

### HPAI H7N9 Viruses

As a newly discovered HPAI subtype with a serious threat to public health and the poultry industry, H7N9 HPAIVs have become a major burden in Asia and raised concerns regarding the potential for a pandemic. 127 They were first detected in Shanghai and Anhui, China in March 2013, 128 129 and subsequently spread to over 20 provinces, including Ningxia, Shaanxi, and Shanxi in 2019.

Studies have shown that these viruses have evolved from low pathogenic variants to highly pathogenic strains, with changes in subtypes and genotypes, increasing their pathogenicity in animal models and expanding their host range to include ferrets and mice. 130 131 The genesis of these H7N9 viruses has been linked to reassortment events between H7 and H9N2 viruses in domestic poultry populations in China. For example, phylogenetic analysis has demonstrated that Tianjin H9N2 isolates belong to the G81 lineage and carry internal genes highly homologous to human H10N8 and H7N9 viruses. 132 Furthermore, the presence of a polybasic amino acid sequence at the HA cleavage site of some H7N9 viruses suggests high pathogenicity in birds, highlighting the need for enhanced surveillance to monitor the spread of these viruses. 133 In addition to their pathogenicity, HPAI H7N9 viruses have been shown to induce severe inflammation in both poultry and humans, leading to high mortality rates. 134 Nationwide vaccination of chickens with an H5/H7 bivalent inactivated avian influenza vaccine since September 2017 has successfully controlled H7N9 avian influenza infections in poultry and prevented human infections. 51 Results have indicated a decreased prevalence of low-pathogenic H7N9 strains, while highly pathogenic H7N9 strains have become dominant since the introduction of the vaccine. 135 Regionally distinct lineages have been established with different genotypes. 136 Phylogenetic analysis has shown that strains from 2019 formed an independent small branch and were genetically distant from strains isolated in 2013 to 2018. 135

Human infections with HPAI H7N9 virus were first detected in March 2013 and are characterized by lower respiratory tract disease, which may progress to severe pneumonia with respiratory failure, multi-organ failure, and death in approximately 40% of reported cases. 137 Furthermore, the detection of virulent mutants of H7N9 in chickens poses an increased threat to human health, emphasizing the importance of controlling and eradicating these viruses to prevent a potential pandemic. 138 Studies of interferon-induced protein 35 (IFI35) have shown that it does not interact with the NS1 encoded by H7N9, resulting in IFI35 playing an opposite virus-enabling role during HPAIV H7N9 infection. 134

Overall, H5 and H7 HPAIVs pose a significant threat to public health and the poultry industry. While the increasing numbers of human infections with HPAIVs have raised concerns of a pandemic threat, the implementation of vaccination has been effective in controlling the spread of the virus in poultry and preventing human infections. Further research is still needed to understand the ecology and evolution of related AIVs to develop effective prevention and control measures.

## The Spread and Transmission of HPAIVs among Species

The transmission dynamics of HPAIVs among avian species and their potential to cross species barriers to humans have been a subject of significant research interest in recent years. Global outbreaks of HPAIVs have been linked to the transmission of the virus from wild migratory birds to domestic poultry, underscoring the role of wild bird populations in the dissemination of AIVs. 97 132 Wild birds, particularly migratory species, play a crucial role in spreading HPAI by carrying the virus over long distances and introducing it to new areas through migration. 139 For instance, the introduction of HPAI H5N1 to North America in 2021 through migratory pathways serves as an example of this pattern. 140 Once introduced, HPAIVs can spread rapidly within domestic bird populations through direct and indirect contact, with infected wild birds transmitting the infection to domestic poultry or contaminating their environments with the virus, thereby facilitating transmission. 141 In addition, the high density and close confinement of domestic poultry make them particularly susceptible to HPAI, exacerbating the spread of the virus within and between flocks. 142 Human activities such as the trade and transportation of poultry and poultry products can also contribute to the spread of HPAI between regions and countries. 143

Studies have shown that wild birds can carry and transmit AIVs, such as H9N2 and H5N8, to other bird species, including domestic poultry and ostriches. 97 144 145 The prevalence of the H9N2 avian virus in wild birds has been investigated, with phylogenetic analysis revealing genetic similarities between avian and human influenza viruses. 146 Moreover, the circulation of HPAIVs in resident wild bird populations in northern Europe during the summer months indicates ongoing transmission within both wild and domestic bird populations. 147 The global dissemination of influenza A viruses, driven by wild bird migration through Arctic and subarctic zones, has been identified as a key factor in the geographic spread of low and HPAIVs. 148 The connectivity of different regions, such as Iceland, to viral flow between the eastern and western hemispheres, has been studied to understand inter-species transmission and reassortment dynamics that influence the spread of AIVs. 149 Understanding the transmission patterns of HPAIVs is crucial for implementing effective surveillance, prevention, and control measures to mitigate the impact of HPAI outbreaks on both wild and domestic bird populations.

HPAIVs primarily spread among avian species through direct contact with contaminated excretions from infected birds, such as respiratory secretions or feces. 150 Additionally, airborne transmission can occur, particularly in densely populated poultry environments, where virus-laden droplets can be dispersed over short distances. 151 Studies have shown that H5N8 HPAIVs have spread globally via migratory waterfowl, with analysis of viral progeny in terrestrial poultry revealing changes in polymerase and accessory genes, indicating potential differences in transmission dynamics between infected waterfowl and terrestrial poultry. 144 Furthermore, the tropism and infectivity of seasonal HPAIVs H5N1 have been studied in ferret models, showing that the HPAI H5N1 virus primarily infects nonciliated cells. 152 Studies have also evaluated the pathogenicity and transmissibility of HPAI H5N1 viruses in different animal models, such as ferrets, demonstrating fatal disease and transmission between co-housed ferrets. 148

Research has also investigated the potential transmission of HPAIVs from wild mammals to domestic mammalian hosts and humans. 153 Genomic analysis has been utilized to reveal the transmission and reassortment of wildlife-borne H5N1 HPAIVs among different hosts, such as birds, pigs, and humans. 154 Furthermore, studies have investigated the airborne transmission of highly pathogenic H7N1 influenza virus in ferrets, indicating the potential for AIVs to become capable of airborne transmission among mammals. 155 Evaluations of the pathogenesis and transmission of novel HPAIVs H5N2 and H5N8 in ferrets and mice have highlighted the potential for cross-species infection. 156

In summary, continued research on different HPAIVs is essential to elucidate their modes of transmission among avian species, mammals, and potentially humans. 157 158 Understanding these transmission dynamics is critical for developing effective prevention and control measures to mitigate the impact of HPAI outbreaks on both animal and human health.

Human infections with avian influenza A viruses are relatively uncommon but can occur sporadically in many countries, typically associated with unprotected exposures to infected poultry or virus-contaminated environments. The primary risk factor for human infection appears to be exposure to infected live or dead poultry or contaminated environments, such as live bird markets. 159 160 Individuals involved in poultry-related activities, such as slaughtering, defeathering, handling carcasses of infected poultry, and preparing poultry for consumption, especially in household settings, may also be at increased risk of exposure and infection with HPAIVs. Additionally, a small number of human infection cases with HPAIVs have been attributed to exposure to infected wild birds, resulting in a wide range of symptoms and complications. 161 162

The pandemic potential of HPAI clade 2.3.4.4 H5 viruses has raised concerns, prompting recommendations for multisectoral collaborations to assess the risk of human-to-human transmissibility and develop countermeasures to prevent disease spread. 147 Qualitative risk assessments conducted in live poultry markets in Dhaka City, Bangladesh, have revealed a high prevalence of HPAIVs, posing a plausible risk of transmission between human and poultry species. 145 Specific mutations in the PB2 and PA proteins have been identified that contribute to the efficient replication of H5N1 influenza viruses in human lung cells, highlighting the importance of multisectoral collaborations to prevent disease spread and assess the likelihood of human-to-human transmission. 147 163 While human-to-human transmission of AIVs is rare, occasional instances of limited nonsustained human-to-human transmission have been reported in a small number of people without poultry exposures. 164 165

Overall, current studies underscore the complex dynamics of HPAIVs and emphasize the need for interdisciplinary collaborations to assess and mitigate the risks associated with transmission among avian species and potentially to humans. The different risk factors highlight the importance of preventive measures, such as proper hygiene practices, personal protective equipment, and surveillance, to mitigate the spread of HPAI among humans. Further research is warranted to enhance our understanding of the modes of transmission of these viruses and to develop effective strategies for prevention and control.

## Prevention and Control Measures of HPAIVs

The widespread dissemination of HPAIVs across multiple continents in recent years has resulted in numerous economically costly poultry outbreaks and posed continuous risks to public health. Additionally, sporadic human infections with HPAIVs have underscored the potential risk of these viruses to cross the species barrier. Given the unprecedented spread of HPAIVs, ongoing poultry outbreaks, human exposures, and the potential for a pandemic, it is imperative to enhance constant surveillance of HPAIVs and implement increased protection measures to safeguard humans from possible infections.

Strategies for controlling HPAI in poultry and wild bird populations encompass several key aspects, including: (1) implementation of surveillance programs targeting high-risk avian populations, such as live bird markets and markets selling poultry, as well as fighting cocks. 166 (2) Conducting global avian influenza surveillance, with a focus on wild birds, poultry, related environments, human cases, and high-risk populations. 167 (3) Development of outbreak management strategies, which may involve rapid response measures, culling infected birds, and implementing biosecurity measures. 168 (4) Providing guidance for site managers of areas where significant numbers of wild birds gather, such as islands, shorelines, and wetlands, to mitigate the risk of HPAI spread. 169 (5) These strategies aim to prevent, detect, and control the spread of HPAI in both poultry and wild bird populations, thereby reducing the risk of transmission to humans and other animals.

Global health initiatives and surveillance programs play a crucial role in rapidly detecting, reporting, and responding to animal outbreaks as the first line of defense. Enhanced risk-based surveillance in animals, as well as surveillance for severe acute respiratory infections and influenza-like illnesses in humans, is essential for early detection and investigation of suspected animal and human cases. This is followed by epidemiological, virological investigations, and genetic sequencing analysis around animal outbreaks and human infections.

Several programs and initiatives facilitate the sharing of information on global or local influenza viruses, for example, the Global Program for Avian Influenza and Human Pandemic Preparedness and Response, which aims to minimize the threat posed to humans by HPAI infection through coordinated global efforts, 170 the Global Influenza Program, which provides strategic guidance, technical support, and coordination of activities essential for combating avian influenza and other influenza viruses, 171 HPAI Surveillance Programs, which include epidemiologic simulation models to study the spread and control of HPAI among commercial and backyard poultry flocks, 167 and the Global Consultation on Highly Pathogenic Avian Influenza by the Food and Agriculture Organization of the United Nations, which focuses on epidemiology, disease surveillance, diagnostic techniques, prevention, control, and risk reduction strategies. 172 Impact of Surveillance and Control studies the effectiveness of ongoing surveillance and control measures on HPAI, emphasizing the global public health risk and economic impact. 172 These initiatives and surveillance programs aim to prevent, detect, and control the spread of HPAI globally, thereby safeguarding both human and animal health.

Vaccination of poultry has emerged as a crucial tool in complementing disease control measures to prevent avian influenza at its source. The effectiveness of poultry vaccination relies on robust surveillance and considerations of local factors such as circulating virus strains, risk assessments, and vaccination implementation conditions. Developing effective vaccines against HPAIVs is essential for public health, with an ideal vaccine aiming to reduce virus spread within and between animal flocks to prevent major outbreaks. Several studies have contributed to advancements in vaccine development against HPAIVs. Kingstad-Bakke et al 173 developed the H5 mosaic (H5M) vaccine antigen, which induced protective levels of humoral immunity against HPAI H5N1 and H5N2 viruses. This research highlights the potential of mosaic-based nanovaccines for avian influenza. Zhang et al 174 discussed the production of high-titer infectious influenza pseudotyped particles with envelope glycoproteins from HPAI H5N1 and avian H7N9 viruses. This experimental approach aids in understanding virus transmission and lethality, emphasizing the importance of vaccine development for epidemic prevention. Wu et al 175 reviewed research progress on human infections with avian influenza H7N9, including key gene mutations, clinical treatment, and vaccine development. Their findings provide a scientific basis for monitoring and preventing H7N9 influenza epidemics. Huang et al 176 assessed the application of a pseudovirus system for re-emerging AIV H5 subtypes in vaccine development. They highlighted the challenges of handling highly pathogenic viruses and the potential of pseudovirus systems in vaccine efficacy and immunogenicity studies. Li and Chen 51 summarized the successful control of H7N9 avian influenza infections in poultry through nationwide vaccination with an H5/H7 bivalent inactivated avian influenza vaccine. This preventive measure not only controlled the virus in poultry but also prevented human infections, demonstrating the importance of vaccination in controlling avian influenza outbreaks.

Overall, the development of effective vaccines against HPAIVs is crucial for preventing potential epidemics and protecting public health. Innovative vaccine technologies, such as mosaic-based nanovaccines and pseudovirus systems, hold promise in enhancing vaccine efficacy and immunogenicity. Additionally, nationwide vaccination efforts have proven successful in controlling avian influenza outbreaks, underscoring the importance of preventive measures in combating infectious diseases. Further research and surveillance are necessary to address the challenges posed by HPAIVs and accelerate vaccine development efforts.

## Conclusion

In conclusion, HPAIVs pose a significant threat to both animal and human health. Continued research and vigilance are paramount to effectively monitor and control these viruses, minimizing their impact on global health and economies. Studies on HPAIVs play a crucial role in understanding their epidemiology, transmission, and virulence, exploring their pandemic potential, and guiding the development of vaccines and antiviral strategies. Additionally, they inform public health policies, surveillance strategies, and response plans to mitigate potential outbreaks.

Given the rapid evolution of HPAIVs, continuous surveillance and risk assessment are critical for controlling outbreaks and reducing impacts on public health and the economy. Early detection of HPAI outbreaks in animals is essential to minimize the risk of transmission to humans and the potential for a pandemic. Furthermore, vigilant field research is necessary to mitigate virological threats to animals raised for food, ensuring food supply security while minimizing transmission risks to humans.

Continued research efforts should focus on exploring passive immunization, assessing pandemic risk associated with HPAI, conducting laboratory studies on altered HPAIVs to understand transmission dynamics, and evaluating various control measures. Mechanistic models used to study HPAI transmission dynamics can contribute to improved strategies for prevention and treatment, ultimately reducing the impact of HPAI on both animal and human health.

## References

[1] Lefkowitz E J Dempsey D M Hendrickson R C Orton R J Siddell S G Smith D B Virus taxonomy: the database of the International Committee on Taxonomy of Viruses (ICTV) Nucleic Acids Res 2018 46(D1):D708 D717 29040670 10.1093/nar/gkx932 PMC5753373

[2] Behboudi S Alphainfluenzavirus influenzaeCABI Compendium.2023; athttps://www.cabidigitallibrary.org/doi/10.1079/cabicompendium.79299

[3] Kim J H Cho C H Shin J H Highly sensitive and label-free detection of influenza H5N1 viral proteins using affinity peptide and porous BSA/MXene nanocomposite electrode Anal Chim Acta 2023 1251 341018 36925304 10.1016/j.aca.2023.341018

[4] Charostad J Rezaei Zadeh Rukerd M Mahmoudvand S A comprehensive review of highly pathogenic avian influenza (HPAI) H5N1: an imminent threat at doorstep Travel Med Infect Dis 2023 55 102638 37652253 10.1016/j.tmaid.2023.102638

[5] Carnegie L Raghwani J Fournié G Hill S C Phylodynamic approaches to studying avian influenza virus Avian Pathol 2023 52 05 289 308 37565466 10.1080/03079457.2023.2236568

[6] Verhagen J H van Dijk J G Vuong O Migratory birds reinforce local circulation of avian influenza viruses PLoS One 2014 9 11 e112366 25391154 10.1371/journal.pone.0112366 PMC4229208

[7] Nuñez I A Ross T M A review of H5Nx avian influenza viruses Ther Adv Vaccines Immunother 2019 7 2.515135518821625E15 10.1177/2515135518821625 PMC6391539 30834359

[8] Kleyheeg E Slaterus R Bodewes R Deaths among wild birds during highly pathogenic avian influenza A (H5N8) virus outbreak, the Netherlands Emerg Infect Dis 2017 23 12 2050 2054 29148372 10.3201/eid2312.171086 PMC5708256

[9] Lean F ZX Vitores A G Reid S M Gross pathology of high pathogenicity avian influenza virus H5N1 2021-2022 epizootic in naturally infected birds in the United Kingdom One Health 2022 14 100392 35686147 10.1016/j.onehlt.2022.100392 PMC9171523

[10] Lai S Qin Y Cowling B J Global epidemiology of avian influenza A H5N1 virus infection in humans, 1997-2015: a systematic review of individual case data Lancet Infect Dis 2016 16 07 e108 e118 27211899 10.1016/S1473-3099(16)00153-5 PMC4933299

[11] Blagodatski A Trutneva K Glazova O Avian influenza in wild birds and poultry: dissemination pathways, monitoring methods, and virus ecology Pathogens 2021 10 05 630 34065291 10.3390/pathogens10050630 PMC8161317

[12] Agüero M Monne I Sánchez A Highly pathogenic avian influenza A(H5N1) virus infection in farmed minks, Spain, October 2022 Euro Surveill 2023 28 03 2.300001E6 10.2807/1560-7917.ES.2023.28.3.2300001 PMC9853945 36695488

[13] Vreman S Kik M Germeraad E Zoonotic mutation of highly pathogenic avian influenza H5N1 virus identified in the brain of multiple wild carnivore species Pathogens 2023 12 02 168 36839440 10.3390/pathogens12020168 PMC9961074

[14] Hampson A W Mackenzie J S The influenza viruses Med J Aust 2006 185(S10):S39 S43 17115950 10.5694/j.1326-5377.2006.tb00705.x

[15] Rimi N A Hassan M Z Chowdhury S A decade of avian influenza in Bangladesh: where are we now? Trop Med Infect Dis 2019 4 03 119 31514405 10.3390/tropicalmed4030119 PMC6789720

[16] Wille M Holmes E C The ecology and evolution of influenza viruses Cold Spring Harb Perspect Med 2020 10 07 a038489 31871237 10.1101/cshperspect.a038489 PMC7328453

[17] Ghedin E Sengamalay N A Shumway M Large-scale sequencing of human influenza reveals the dynamic nature of viral genome evolution Nature 2005 437 7062 1162 1166 16208317 10.1038/nature04239

[18] Bouvier N M Palese P The biology of influenza viruses Vaccine 2008 26(Suppl 4, Suppl 4):D49 D53 19230160 10.1016/j.vaccine.2008.07.039 PMC3074182

[19] Kosik I Yewdell J W Influenza hemagglutinin and neuraminidase: Yin–Yang proteins coevolving to thwart immunity Viruses 2019 11 04 346 31014029 10.3390/v11040346 PMC6520700

[20] Kayal V Brundha M Sivaswamy V New concepts of antigenic shift and antigenic drift in influenza and corona-a review of literature Int J Pharmaceutical Res 2021 13 01 9.752366E6

[21] Yoon S W Webby R J Webster R G Evolution and ecology of influenza A viruses Curr Top Microbiol Immunol 2014 385 359 375 24990620 10.1007/82_2014_396

[22] Blaurock C Scheibner D Landmann M Non-basic amino acids in the hemagglutinin proteolytic cleavage site of a European H9N2 avian influenza virus modulate virulence in turkeys Sci Rep 2020 10 01 21226 33277593 10.1038/s41598-020-78210-8 PMC7718272

[23] Verhagen J H Eriksson P Leijten L Host range of influenza A virus H1 to H16 in Eurasian ducks based on tissue and receptor binding studies J Virol 2021 95 06 1873 20 10.1128/JVI.01873-20 PMC8094940 33361418

[24] World Health Organization Antigenic and genetic characteristics of zoonotic influenza A viruses and development of candidate vaccine viruses for pandemic preparedness Wkly Epidemiol Rec 2020 95 44 525 539

[25] WHO/OIE/FAO H5N1 Evolution Working Group Continued evolution of highly pathogenic avian influenza A (H5N1): updated nomenclature Influenza Other Respir Viruses 2012 6 01 1 5 22035148 10.1111/j.1750-2659.2011.00298.x PMC5074649

[26] Neumann G Chen H Gao G F Shu Y Kawaoka Y H5N1 influenza viruses: outbreaks and biological properties Cell Res 2010 20 01 51 61 19884910 10.1038/cr.2009.124 PMC2981148

[27] Bi Y Chen Q Wang Q Genesis, evolution and prevalence of H5N6 avian influenza viruses in China Cell Host Microbe 2016 20 06 810 821 27916476 10.1016/j.chom.2016.10.022

[28] Du Y Chen M Yang J Molecular evolution and emergence of H5N6 avian influenza virus in central China J Virol 2017 91 12 143 17 10.1128/JVI.00143-17 PMC5446651 28404845

[29] Cui Y Li Y Li M Evolution and extensive reassortment of H5 influenza viruses isolated from wild birds in China over the past decade Emerg Microbes Infect 2020 9 01 1793 1803 32686602 10.1080/22221751.2020.1797542 PMC7473172

[30] Gu W Shi J Cui P Novel H5N6 reassortants bearing the clade 2.3.4.4b HA gene of H5N8 virus have been detected in poultry and caused multiple human infections in China Emerg Microbes Infect 2022 11 01 1174 1185 35380505 10.1080/22221751.2022.2063076 PMC9126593

[31] Hesterberg U Harris K Stroud D Avian influenza surveillance in wild birds in the European Union in 2006 Influenza Other Respir Viruses 2009 3 01 1 14 19453436 10.1111/j.1750-2659.2008.00058.x PMC4941908

[32] Adlhoch C Gossner C Koch G Comparing introduction to Europe of highly pathogenic avian influenza viruses A(H5N8) in 2014 and A(H5N1) in 2005 Euro Surveill 2014 19 50 20996 25597538 10.2807/1560-7917.es2014.19.50.20996

[33] Olsen B Munster V J Wallensten A Waldenström J Osterhaus A D Fouchier R A Global patterns of influenza a virus in wild birds Science 2006 312 5772 384 388 16627734 10.1126/science.1122438

[34] European Food Safety Authority, European Centre for Disease Prevention and Control Highly pathogenic avian influenza A subtype H5N8 EFSA J 2014 12 12 3941

[35] Jeong J Kang H M Lee E K Highly pathogenic avian influenza virus (H5N8) in domestic poultry and its relationship with migratory birds in South Korea during 2014 Vet Microbiol 2014 173(3–4):249 257 25192767 10.1016/j.vetmic.2014.08.002

[36] Lee D H Torchetti M K Winker K Ip H S Song C S Swayne D E Intercontinental spread of Asian-origin H5N8 to North America through Beringia by migratory birds J Virol 2015 89 12 6521 6524 25855748 10.1128/JVI.00728-15 PMC4474297

[37] Li Y Li M Li Y Outbreaks of highly pathogenic avian influenza (H5N6) virus subclade 2.3.4.4 h in swans, Xinjiang, Western China, 2020 Emerg Infect Dis 2020 26 12 2956 2960 33030424 10.3201/eid2612.201201 PMC7706961

[38] Youk S Torchetti M K Lantz K H5N1 highly pathogenic avian influenza clade 2.3.4.4b in wild and domestic birds: Introductions into the United States and reassortments, December 2021-April 2022 Virology 2023 587 109860 37572517 10.1016/j.virol.2023.109860

[39] Howley P M Knipe D M Fields Virology: Emerging Viruses Philadelphia, PA: Lippincott Williams & Wilkins; 2020

[40] Centers for Disease Control and Prevention.Reported human infections with avian influenza A viruses 2023. Accessed 1 March 2024 at:https://www.cdc.gov/flu/avianflu/reported-human-infections.htm

[41] Liu Q Liu D Y Yang Z Q Characteristics of human infection with avian influenza viruses and development of new antiviral agents Acta Pharmacol Sin 2013 34 10 1257 1269 24096642 10.1038/aps.2013.121 PMC3791557

[42] Government of Canada.Biosafety directive for new and emerging influenza A virusesAccessed 1 March 2024 at:https://www.canada.ca/en/public-health/services/laboratory-biosafety-biosecurity/biosafety-directives-advisories-notifications/new-emerging-influenza-a-viruses.html

[43] Mostafa A Abdelwhab E M Mettenleiter T C Pleschka S Zoonotic potential of influenza A viruses: a comprehensive overview Viruses 2018 10 09 497 30217093 10.3390/v10090497 PMC6165440

[44] Lycett S J Duchatel F Digard P A brief history of bird flu Philos Trans R Soc Lond B Biol Sci 2019 374 1775 2.0180257E7 10.1098/rstb.2018.0257 PMC6553608 31056053

[45] Centers for Disease Control and Prevention.Highlights in the History of Avian Influenza (Bird Flu)Accessed 1 March 2024 at:https://www.cdc.gov/flu/avianflu/timeline/avian-timeline-background.htm

[46] Hu T Zhao H Zhang Y Fatal influenza A (H5N1) virus infection in zoo-housed Tigers in Yunnan Province, China Sci Rep 2016 6 01 25845 27162026 10.1038/srep25845 PMC4861906

[47] Keawcharoen J Oraveerakul K Kuiken T Avian influenza H5N1 in tigers and leopards Emerg Infect Dis 2004 10 12 2189 2191 15663858 10.3201/eid1012.040759 PMC3323383

[48] World Organisation for Animal Health.Immediate notification highly pathogenic influenza A viruses (Inf. with) (non‐poultry including wild birds) 2017‐), Estonia. Accessed1 March 2024 at:https://www.woah.org/en/disease/avian-influenza/#ui-id-2

[49] World Health Organization.Cumulative number of confirmed human cases for avian influenza A(H5N1) reported to WHO,2003–2023. Accessed 1 March 2024 at:https://www.who.int/publications/m/item/cumulative-number-of-confirmed-human-cases-for-avian-influenza-a(h5n1)-reported-to-who-2003-2022-5-jan-2023

[50] Dalli J. Commission Decision 2010/367/EU of 25 June 2010 on the implementation by Member States of surveillance programmes for avian influenza in poultry and wild birds Official Journal of the European Union 2010 166 22 32

[51] Li C Chen H H7N9 influenza virus in China Cold Spring Harb Perspect Med 2021 11 08 a038349 32205415 10.1101/cshperspect.a038349 PMC8327827

[52] Huang P Sun L Li J Potential cross-species transmission of highly pathogenic avian influenza H5 subtype (HPAI H5) viruses to humans calls for the development of H5-specific and universal influenza vaccines Cell Discov 2023 9 01 58 37328456 10.1038/s41421-023-00571-x PMC10275984

[53] Tian H Zhou S Dong L Avian influenza H5N1 viral and bird migration networks in Asia Proc Natl Acad Sci U S A 2015 112 01 172 177 25535385 10.1073/pnas.1405216112 PMC4291667

[54] Bragstad K Jørgensen P H Handberg K Hammer A S Kabell S Fomsgaard A First introduction of highly pathogenic H5N1 avian influenza A viruses in wild and domestic birds in Denmark, Northern Europe Virol J 2007 4 43 17498292 10.1186/1743-422X-4-43 PMC1876802

[55] Lewis N S Banyard A C Whittard E Emergence and spread of novel H5N8, H5N5 and H5N1 clade 2.3.4.4 highly pathogenic avian influenza in 2020 Emerg Microbes Infect 2021 10 01 148 151 33400615 10.1080/22221751.2021.1872355 PMC7832535

[56] European Food Safety Authority, European Centre for Disease Prevention, Control, European Union Reference Laboratory for Avian Influenza Adlhoch C Fusaro A Gonzales J L Avian influenza overview September - December 2021 EFSA J 2021 19 12 e07108 34987626 10.2903/j.efsa.2021.7108 PMC8698678

[57] Caliendo V Lewis N S Pohlmann A Transatlantic spread of highly pathogenic avian influenza H5N1 by wild birds from Europe to North America in 2021 Sci Rep 2022 12 01 11729 35821511 10.1038/s41598-022-13447-z PMC9276711

[58] Günther A Krone O Svansson V Iceland as stepping stone for spread of highly pathogenic avian influenza virus between Europe and North America Emerg Infect Dis 2022 28 12 2383 2388 36261139 10.3201/eid2812.221086 PMC9707596

[59] USDA APHIS.Confirmations of highly pathogenic avian influenza in commercial and backyard flocksAccessed 1 March 2024 at:https://www.aphis.usda.gov/livestock-poultry-disease/avian/avian-influenza/hpai-detections/commercial-backyard-flocks

[60] Elsmo E Wünschmann A Beckmen K Pathology of natural infection with highly pathogenic avian influenza virus (H5N1) clade 2.3.4.4 b in wild terrestrial mammals in the United States in 2022 BioRxiv 2023 2023.03 10.3201/eid2912.230464 PMC10683806 37987580

[61] USDA APHIS.Detections of highly pathogenic avian influenza in mammalsAccessed 1 March 2024 at:https://www.aphis.usda.gov/livestock-poultry-disease/avian/avian-influenza/hpai-detections/mammals

[62] Rijks J M Hesselink H Lollinga P Highly pathogenic avian influenza A (H5N1) virus in wild red foxes, the Netherlands, 2021 Emerg Infect Dis 2021 27 11 2960 2962 34670656 10.3201/eid2711.211281 PMC8544991

[63] European Food Safety Authority European Centre for Disease Prevention and Control European Union Reference Laboratory for Avian Influenza Adlhoch C Fusaro A Gonzales J L Avian influenza overview June-September 2023 EFSA J 2023 21 10 e08328 37809353 10.2903/j.efsa.2023.8328 PMC10552073

[64] Fisheries N Recent increase in seal deaths in maine linked to avian fluAccessed 1 March 2024 at:https://www.fisheries.noaa.gov/feature-story/recent-increase-seal-deaths-maine-linked-avian-flu

[65] European Food Safety Authority European Centre for Disease Prevention and Control European Union Reference Laboratory for Avian Influenza Adlhoch C Fusaro A Gonzales J L Avian influenza overview December 2021 - March 2022 EFSA J 2022 20 04 e07289 35386927 10.2903/j.efsa.2022.7289 PMC8978176

[66] Oliver I Roberts J Brown C S A case of avian influenza A(H5N1) in England, January 2022 Euro Surveill 2022 27 05 2.200061E6 10.2807/1560-7917.ES.2022.27.5.2200061 PMC8815099 35115075

[67] Qu N Zhao B Chen Z Genetic characteristics, pathogenicity and transmission of H5N6 highly pathogenic avian influenza viruses in Southern China Transbound Emerg Dis 2019 66 06 2411 2425 31328387 10.1111/tbed.13299

[68] Li H Li Q Li B Continuous reassortment of clade 2.3. 4.4 H5N6 highly pathogenetic avian influenza viruses demonstrating high risk to public health Pathogens 2020 9 08 670 32824873 10.3390/pathogens9080670 PMC7460007

[69] Zhang J Ye H Liu Y Liao M Qi W Resurgence of H5N6 avian influenza virus in 2021 poses new threat to public health Lancet Microbe 2022 3 08 e558 35750068 10.1016/S2666-5247(22)00148-3

[70] Shittu I Bianco A Gado D First detection of highly pathogenic H5N6 avian influenza virus on the African continent Emerg Microbes Infect 2020 9 01 886 888 32312185 10.1080/22221751.2020.1757999 PMC7241522

[71] Yang L Zhu W Li X Genesis and dissemination of highly pathogenic H5N6 avian influenza viruses J Virol 2017 91 05 2199 16 10.1128/JVI.02199-16 PMC5309950 28003485

[72] Zhang Y Chen M Huang Y Human infections with novel reassortant H5N6 avian influenza viruses in China Emerg Microbes Infect 2017 6 06 e50 28588293 10.1038/emi.2017.38 PMC5520314

[73] Sun W Li J Hu J Genetic analysis and biological characteristics of different internal gene origin H5N6 reassortment avian influenza virus in China in 2016 Vet Microbiol 2018 219 200 211 29778197 10.1016/j.vetmic.2018.04.023

[74] Zhang R Lei Z Liu C Live poultry feeding and trading network and the transmission of avian influenza A(H5N6) virus in a large city in China, 2014-2015 Int J Infect Dis 2021 108 72 80 34000420 10.1016/j.ijid.2021.05.022

[75] Zhu W Li X Dong J Epidemiologic, clinical, and genetic characteristics of human infections with influenza A (H5N6) viruses, China Emerg Infect Dis 2022 28 07 1332 1344 35476714 10.3201/eid2807.212482 PMC9239879

[76] Sun H Pu J Wei Y Highly pathogenic avian influenza H5N6 viruses exhibit enhanced affinity for human type sialic acid receptor and in-contact transmission in model ferrets J Virol 2016 90 14 6235 6243 27122581 10.1128/JVI.00127-16 PMC4936137

[77] Pan M Gao R Lv Q Human infection with a novel, highly pathogenic avian influenza A (H5N6) virus: virological and clinical findings J Infect 2016 72 01 52 59 26143617 10.1016/j.jinf.2015.06.009

[78] Yang L Zhao X Li X Case report for human infection with a highly pathogenic avian influenza A (H5N6) virus in Beijing, China 2019 Biosafety Health 2020 2 01 49 52

[79] Lee Y J Kang H M Lee E K Novel reassortant influenza A(H5N8) viruses, South Korea, 2014 Emerg Infect Dis 2014 20 06 1087 1089 24856098 10.3201/eid2006.140233 PMC4036756

[80] European Food Safety Authority European Centre for Disease Prevention and Control and European Union Reference Laboratory for Avian Influenza Adlhoch C Fusaro A Kuiken T Avian influenza overview February - May 2020 EFSA J 2020 18 06 e06194 32874346 10.2903/j.efsa.2020.6194 PMC7448026

[81] Xiong J Zhou H Fan L Emerging highly pathogenic avian influenza (H5N8) virus in migratory birds in Central China, 2020 Emerg Microbes Infect 2021 10 01 1503 1506 34260340 10.1080/22221751.2021.1956372 PMC8330791

[82] Floyd T Banyard A C Lean F ZX Encephalitis and death in wild mammals at a rehabilitation center after infection with highly pathogenic avian influenza A(H5N8) virus, United Kingdom Emerg Infect Dis 2021 27 11 2856 2863 34670647 10.3201/eid2711.211225 PMC8544989

[83] King J Harder T Globig A Highly pathogenic avian influenza virus incursions of subtype H5N8, H5N5, H5N1, H5N4, and H5N3 in Germany during 2020-21 Virus Evol 2022 8 01 veac035 35478715 10.1093/ve/veac035 PMC9037367

[84] Liang Y Nissen J N Krog J S Novel clade 2.3.4.4 b highly pathogenic avian influenza A H5N8 and H5N5 viruses in Denmark, 2020 Viruses 2021 13 05 886 34065033 10.3390/v13050886 PMC8151437

[85] Pyankova O G Susloparov I M Moiseeva A A Isolation of clade 2.3.4.4b A(H5N8), a highly pathogenic avian influenza virus, from a worker during an outbreak on a poultry farm, Russia, December 2020 Euro Surveill 2021 26 24 2.100439E6 10.2807/1560-7917.ES.2021.26.24.2100439 PMC8212591 34142650

[86] Hall J S Dusek R J Spackman E Rapidly expanding range of highly pathogenic avian influenza viruses Emerg Infect Dis 2015 21 07 1251 1252 26079209 10.3201/eid2107.150403 PMC4480408

[87] Global Consortium for H5N8 and Related Influenza Viruses Lycett S J Pohlmann A Staubach C Genesis and spread of multiple reassortants during the 2016/2017 H5 avian influenza epidemic in Eurasia Proc Natl Acad Sci U S A 2020 117 34 20814 20825 32769208 10.1073/pnas.2001813117 PMC7456104

[88] Fusaro A Zecchin B Vrancken B Disentangling the role of Africa in the global spread of H5 highly pathogenic avian influenza Nat Commun 2019 10 01 5310 31757953 10.1038/s41467-019-13287-y PMC6874648

[89] Świętoń E Fusaro A Shittu I Sub-Saharan Africa and Eurasia ancestry of reassortant highly pathogenic avian influenza A(H5N8) virus, Europe, December 2019 Emerg Infect Dis 2020 26 07 1557 1561 32568059 10.3201/eid2607.200165 PMC7323556

[90] Tian J Bai X Li M Highly pathogenic avian influenza virus (H5N1) clade 2.3.4.4 b introduced by wild birds, China, 2021 Emerg Infect Dis 2023 29 07 1367 1375 37347504 10.3201/eid2907.221149 PMC10310395

[91] Śmietanka K Świętoń E Kozak E Highly pathogenic avian influenza H5N8 in Poland in 2019–2020 J Vet Res (Pulawy) 2020 64 04 469 476 10.2478/jvetres-2020-0078 PMC7734677 33367134

[92] Sobolev I Sharshov K Dubovitskiy N Highly pathogenic avian influenza A(H5N8) virus clade 2.3.4.4 b, Western Siberia, Russia, 2020 Emerg Infect Dis 2021 27 08 2224 2227 34287138 10.3201/eid2708.204969 PMC8314819

[93] Beerens N Heutink R Harders F Incursion of novel highly pathogenic avian influenza A(H5N8) virus, the Netherlands, October 2020 Emerg Infect Dis 2021 27 06 1750 1753 34013854 10.3201/eid2706.204464 PMC8153856

[94] Isoda N Twabela A T Bazarragchaa E Re-invasion of H5N8 high pathogenicity avian influenza virus clade 2.3. 4.4 b in Hokkaido, Japan, 2020 Viruses 2020 12 12 1439 33327524 10.3390/v12121439 PMC7764937

[95] Jeong S Lee D-H Kwon J-H Highly pathogenic avian influenza clade 2.3.4.4 b subtype H5N8 virus isolated from Mandarin duck in South Korea, 2020 Viruses 2020 12 12 1389 33291548 10.3390/v12121389 PMC7761861

[96] Baek Y G Lee Y N Lee D H Multiple reassortants of H5N8 clade 2.3.4.4 b highly pathogenic avian influenza viruses detected in South Korea during the Winter of 2020–2021 Viruses 2021 13 03 490 33809549 10.3390/v13030490 PMC8001867

[97] Beerens N Germeraad E A Venema S Verheij E Pritz-Verschuren S BE Gonzales J L Comparative pathogenicity and environmental transmission of recent highly pathogenic avian influenza H5 viruses Emerg Microbes Infect 2021 10 01 97 108 33350337 10.1080/22221751.2020.1868274 PMC7832006

[98] Khalil A M Fujimoto Y Kojima I Genetic characterization of H5N8 highly pathogenic avian influenza viruses isolated from falcated ducks and environmental water in Japan in November 2020 Pathogens 2021 10 02 171 33557405 10.3390/pathogens10020171 PMC7915289

[99] Lopez-Martinez I Balish A Barrera-Badillo G Highly pathogenic avian influenza A(H7N3) virus in poultry workers, Mexico, 2012 Emerg Infect Dis 2013 19 09 1531 1534 23965808 10.3201/eid1909.130087 PMC3810917

[100] Shu Y McCauley J GISAID: Global initiative on sharing all influenza data - from vision to reality Euro Surveill 2017 22 13 30494 28382917 10.2807/1560-7917.ES.2017.22.13.30494 PMC5388101

[101] Navarro-Lopez R Xu W Gomez-Romero N Velazquez-Salinas L Berhane Y Phylogenetic inference of the 2022 highly pathogenic H7N3 avian influenza outbreak in Northern Mexico Pathogens 2022 11 11 1284 36365034 10.3390/pathogens11111284 PMC9692817

[102] Youk S Lee D-H Killian M L Pantin-Jackwood M J Swayne D E Torchetti M K Highly pathogenic avian influenza A (H7N3) virus in poultry, United States, 2020 Emerg Infect Dis 2020 26 12 2966 2969 33030423 10.3201/eid2612.202790 PMC7706930

[103] Lu L Lycett S J Leigh Brown A J Determining the phylogenetic and phylogeographic origin of highly pathogenic avian influenza (H7N3) in Mexico PLoS One 2014 9 09 e107330 25226523 10.1371/journal.pone.0107330 PMC4165766

[104] Youk S Lee D H Ferreira H L Rapid evolution of Mexican H7N3 highly pathogenic avian influenza viruses in poultry PLoS One 2019 14 09 e0222457 31513638 10.1371/journal.pone.0222457 PMC6742402

[105] Trovão N S Talavera G A Nelson M I Perez de la Rosa J D Evolution of highly pathogenic H7N3 avian influenza viruses in Mexico Zoonoses Public Health 2020 67 03 318 323 31912652 10.1111/zph.12673

[106] Youk S S Lee D H Leyson C M Loss of fitness of Mexican H7N3 highly pathogenic avian influenza virus in mallards after circulating in chickens J Virol 2019 93 14 543 19 10.1128/JVI.00543-19 PMC6600187 31068421

[107] Beerens N Heutink R Harders F Bossers A Koch G Peeters B Emergence and selection of a highly pathogenic avian influenza H7N3 virus J Virol 2020 94 08 1818 1819 10.1128/JVI.01818-19 PMC7108855 31969434

[108] Banks J Speidel E S Moore E Changes in the haemagglutinin and the neuraminidase genes prior to the emergence of highly pathogenic H7N1 avian influenza viruses in Italy Arch Virol 2001 146 05 963 973 11448033 10.1007/s007050170128

[109] Röhm C Horimoto T Kawaoka Y Süss J Webster R G Do hemagglutinin genes of highly pathogenic avian influenza viruses constitute unique phylogenetic lineages? Virology 1995 209 02 664 670 7778300 10.1006/viro.1995.1301

[110] Navarro-López R Solís-Hernández M Márquez-Ruiz M A Epizootic of highly pathogenic H7N3 Avian Influenza in an ecologic reserve in Mexico bioRxiv 2020 2020.03. 05.978502

[111] Lee D H Criado M F Swayne D E Pathobiological origins and evolutionary history of highly pathogenic avian influenza viruses Cold Spring Harb Perspect Med 2021 11 02 a038679 31964650 10.1101/cshperspect.a038679 PMC7849344

[112] Kayed A E Kutkat O Kandeil A Comparative pathogenic potential of avian influenza H7N3 viruses isolated from wild birds in Egypt and their sensitivity to commercial antiviral drugs Arch Virol 2023 168 03 82 36757481 10.1007/s00705-022-05646-w PMC9909137

[113] Skowronski D M Li Y Tweed S A Protective measures and human antibody response during an avian influenza H7N3 outbreak in poultry in British Columbia, Canada CMAJ 2007 176 01 47 53 17200390 10.1503/cmaj.060204 PMC1764568

[114] Trinh T TT Tiwari I Durairaj K Genetic characterization and pathogenesis of avian influenza virus H7N3 isolated from spot-billed ducks in South Korea, early 2019 Viruses 2021 13 05 856 34067187 10.3390/v13050856 PMC8151380

[115] Süss J Schäfer J Sinnecker H Webster R G Influenza virus subtypes in aquatic birds of eastern Germany Arch Virol 1994 135(1–2):101 114 8198436 10.1007/BF01309768

[116] Scheibner D Blaurock C Mettenleiter T C Abdelwhab E M Virulence of three European highly pathogenic H7N1 and H7N7 avian influenza viruses in Pekin and Muscovy ducks BMC Vet Res 2019 15 01 142 31077209 10.1186/s12917-019-1899-4 PMC6511205

[117] Spruit C M Zhu X Tomris I N-glycolylneuraminic acid binding of avian and equine H7 influenza A viruses J Virol 2022 96 05 e0212021 35044215 10.1128/jvi.02120-21 PMC8906439

[118] van der Goot J A Koch G de Jong M C van Boven M Quantification of the effect of vaccination on transmission of avian influenza (H7N7) in chickens Proc Natl Acad Sci U S A 2005 102 50 18141 18146 16330777 10.1073/pnas.0505098102 PMC1312373

[119] Park M S Steel J García-Sastre A Swayne D Palese P Engineered viral vaccine constructs with dual specificity: avian influenza and Newcastle disease Proc Natl Acad Sci U S A 2006 103 21 8203 8208 16717196 10.1073/pnas.0602566103 PMC1464378

[120] DeLay P D Casey H L Tubiash H S Comparative study of fowl plague virus and a virus isolated from man Public Health Rep 1967 82 07 615 620 4291102 PMC1920013

[121] Kurtz J Manvell R J Banks J Avian influenza virus isolated from a woman with conjunctivitis Lancet 1996 348 9031 901 902 8826845 10.1016/S0140-6736(05)64783-6

[122] Fouchier R A Schneeberger P M Rozendaal F W Avian influenza A virus (H7N7) associated with human conjunctivitis and a fatal case of acute respiratory distress syndrome Proc Natl Acad Sci U S A 2004 101 05 1356 1361 14745020 10.1073/pnas.0308352100 PMC337057

[123] Influenza Task Force Puzelli S Rossini G Facchini M Human infection with highly pathogenic A(H7N7) avian influenza virus, Italy, 2013 Emerg Infect Dis 2014 20 10 1745 1749 25271444 10.3201/eid2010.140512 PMC4193179

[124] Munster V J de Wit E van Riel D The molecular basis of the pathogenicity of the Dutch highly pathogenic human influenza A H7N7 viruses J Infect Dis 2007 196 02 258 265 17570113 10.1086/518792

[125] Belser J A Blixt O Chen L M Contemporary North American influenza H7 viruses possess human receptor specificity: Implications for virus transmissibility Proc Natl Acad Sci U S A 2008 105 21 7558 7563 18508975 10.1073/pnas.0801259105 PMC2396559

[126] Chan M C Chan R W Chan L L Tropism and innate host responses of a novel avian influenza A H7N9 virus: an analysis of ex-vivo and in-vitro cultures of the human respiratory tract Lancet Respir Med 2013 1 07 534 542 24461614 10.1016/S2213-2600(13)70138-3 PMC7164816

[127] Song C Liu Y Jiang X Ultrasensitive SERS determination of avian influenza A H7N9 virus via exonuclease III-assisted cycling amplification Talanta 2019 205 120137 31450475 10.1016/j.talanta.2019.120137

[128] Chen Y Liang W Yang S Human infections with the emerging avian influenza A H7N9 virus from wet market poultry: clinical analysis and characterisation of viral genome Lancet 2013 381 9881 1916 1925 23623390 10.1016/S0140-6736(13)60903-4 PMC7134567

[129] Gao R Cao B Hu Y Human infection with a novel avian-origin influenza A (H7N9) virus N Engl J Med 2013 368 20 1888 1897 23577628 10.1056/NEJMoa1304459

[130] Ma M J Yang Y Fang L Q Highly pathogenic avian H7N9 influenza viruses: recent challenges Trends Microbiol 2019 27 02 93 95 30553653 10.1016/j.tim.2018.11.008

[131] Li G Wang X Li Q Development of an immunochromatographic strip for rapid detection of H7 subtype avian influenza viruses Virol J 2021 18 01 68 33827632 10.1186/s12985-021-01537-9 PMC8025375

[132] Zhang X Li Y Jin S H9N2 influenza virus spillover into wild birds from poultry in China bind to human-type receptors and transmit in mammals via respiratory droplets Transbound Emerg Dis 2022 69 02 669 684 33566453 10.1111/tbed.14033

[133] Qi W Jia W Liu D Emergence and adaptation of a novel highly pathogenic H7N9 influenza virus in birds and humans from a 2013 human-infecting low-pathogenic ancestor J Virol 2018 92 02 921 17 10.1128/JVI.00921-17 PMC5752946 29070694

[134] Yang H Winkler W Wu X Interferon Inducer IFI35 regulates RIG-I-mediated innate antiviral response through mutual antagonism with Influenza protein NS1 J Virol 2021 95 11 283 21 10.1128/JVI.00283-21 PMC8139692 33692214

[135] Wu Y Hu J Jin X Accelerated evolution of H7N9 subtype influenza virus under vaccination pressure Virol Sin 2021 36 05 1124 1132 33974230 10.1007/s12250-021-00383-x PMC8112217

[136] Zhu H Lam T TY Smith D K Guan Y Emergence and development of H7N9 influenza viruses in China Curr Opin Virol 2016 16 106 113 26922715 10.1016/j.coviro.2016.01.020

[137] Li Q Zhou L Zhou M Epidemiology of human infections with avian influenza A(H7N9) virus in China N Engl J Med 2014 370 06 520 532 23614499 10.1056/NEJMoa1304617 PMC6652192

[138] Shi J Deng G Kong H H7N9 virulent mutants detected in chickens in China pose an increased threat to humans Cell Res 2017 27 12 1409 1421 29151586 10.1038/cr.2017.129 PMC5717404

[139] Keawcharoen J van den Broek J Bouma A Tiensin T Osterhaus A D Heesterbeek H Wild birds and increased transmission of highly pathogenic avian influenza (H5N1) among poultry, Thailand Emerg Infect Dis 2011 17 06 1016 1022 21749762 10.3201/eid1706.100880 PMC3358188

[140] Harvey J A Mullinax J M Runge M C Prosser D J The changing dynamics of highly pathogenic avian influenza H5N1: Next steps for management & science in North America Biol Conserv 2023 282 110041

[141] Tsiodras S Kelesidis T Kelesidis I Bauchinger U Falagas M E Human infections associated with wild birds J Infect 2008 56 02 83 98 18096237 10.1016/j.jinf.2007.11.001 PMC7172416

[142] Chowdhury S Hossain M E Ghosh P K The pattern of highly pathogenic avian influenza H5N1 outbreaks in South Asia Trop Med Infect Dis 2019 4 04 138 31783701 10.3390/tropicalmed4040138 PMC6958390

[143] Wu T Perrings C The live poultry trade and the spread of highly pathogenic avian influenza: regional differences between Europe, West Africa, and Southeast Asia PLoS One 2018 13 12 e0208197 30566454 10.1371/journal.pone.0208197 PMC6300203

[144] Puranik A Slomka M J Warren C J Transmission dynamics between infected waterfowl and terrestrial poultry: Differences between the transmission and tropism of H5N8 highly pathogenic avian influenza virus (clade 2.3.4.4a) among ducks, chickens and turkeys Virology 2020 541 113 123 32056709 10.1016/j.virol.2019.10.014

[145] Islam S S Akwar H Hossain M M Qualitative risk assessment of transmission pathways of highly pathogenic avian influenza (HPAI) virus at live poultry markets in Dhaka city, Bangladesh Zoonoses Public Health 2020 67 06 658 672 32558220 10.1111/zph.12746

[146] Dong G Luo J Zhang H Phylogenetic diversity and genotypical complexity of H9N2 influenza A viruses revealed by genomic sequence analysis PLoS One 2011 6 02 e17212 21386964 10.1371/journal.pone.0017212 PMC3046171

[147] Yamaji R Saad M D Davis C T Pandemic potential of highly pathogenic avian influenza clade 2.3.4.4 A(H5) viruses Rev Med Virol 2020 30 03 e2099 32135031 10.1002/rmv.2099 PMC9285678

[148] Pulit-Penaloza J A Brock N Pappas C Characterization of highly pathogenic avian influenza H5Nx viruses in the ferret model Sci Rep 2020 10 01 12700 32728042 10.1038/s41598-020-69535-5 PMC7391700

[149] Gass J D Jr Dusek R J Hall J S Global dissemination of influenza A virus is driven by wild bird migration through arctic and subarctic zones Mol Ecol 2023 32 01 198 213 36239465 10.1111/mec.16738 PMC9797457

[150] Feare C J Role of wild birds in the spread of highly pathogenic avian influenza virus H5N1 and implications for global surveillance Avian Dis 2010 54 01 201 212 20521633 10.1637/8766-033109-ResNote.1

[151] Bertran K Balzli C Kwon Y-K Tumpey T M Clark A Swayne D E Airborne transmission of highly pathogenic influenza virus during processing of infected poultry Emerg Infect Dis 2017 23 11 1806 1814 29047426 10.3201/eid2311.170672 PMC5652435

[152] Zeng H Goldsmith C S Kumar A Tropism and infectivity of a seasonal A (H1N1) and a highly pathogenic avian A (H5N1) influenza virus in primary differentiated ferret nasal epithelial cell cultures J Virol 2019 93 10 80 19 10.1128/JVI.00080-19 PMC6498040 30814288

[153] Ortiz J R Katz M A Mahmoud M N Lack of evidence of avian-to-human transmission of avian influenza A (H5N1) virus among poultry workers, Kano, Nigeria, 2006 J Infect Dis 2007 196 11 1685 1691 18008254 10.1086/522158

[154] Lei F Shi W Prospective of genomics in revealing transmission, reassortment and evolution of wildlife-borne avian influenza A (H5N1) viruses Curr Genomics 2011 12 07 466 474 22547954 10.2174/138920211797904052 PMC3219842

[155] Sutton T C Finch C Shao H Airborne transmission of highly pathogenic H7N1 influenza virus in ferrets J Virol 2014 88 12 6623 6635 24696487 10.1128/JVI.02765-13 PMC4054360

[156] Pulit-Penaloza J A Brock N Belser J A Highly pathogenic avian influenza A(H5N1) virus of clade 2.3.4.4b isolated from a human case in Chile causes fatal disease and transmits between co-housed ferrets Emerg Microbes Infect 2024 13 12 2.332667E6 10.1080/22221751.2024.2332667 PMC11177717 38494746

[157] Xu W Dai Y Hua C Genomic signature analysis of the recently emerged highly pathogenic A(H5N8) avian influenza virus: implying an evolutionary trend for bird-to-human transmission Microbes Infect 2017 19 12 597 604 28889970 10.1016/j.micinf.2017.08.006

[158] European Food Safety Authority European Centre for Disease Prevention and Control and European Union Reference Laboratory for Avian Influenza Adlhoch C Fusaro A Kuiken T Avian influenza overview November 2019- February2020 EFSA J 2020 18 03 e06096 32874270 10.2903/j.efsa.2020.6096 PMC7448010

[159] Zhou L Liao Q Dong L Risk factors for human illness with avian influenza A (H5N1) virus infection in China J Infect Dis 2009 199 12 1726 1734 19416076 10.1086/599206 PMC2759027

[160] Ly S Vong S Cavailler P Environmental contamination and risk factors for transmission of highly pathogenic avian influenza A(H5N1) to humans, Cambodia, 2006-2010 BMC Infect Dis 2016 16 01 631 27809855 10.1186/s12879-016-1950-z PMC5095992

[161] Shi J Zeng X Cui P Yan C Chen H Alarming situation of emerging H5 and H7 avian influenza and effective control strategies Emerg Microbes Infect 2023 12 01 2.155072E6 10.1080/22221751.2022.2155072 PMC9754034 36458831

[162] Sun Z Li Y An Q Gao X Wang H Risk factors contributing to highly pathogenic avian influenza H5N6 in China, 2014 Based on a MaxEnt model Transbound Emerg Dis 2021 2023 2023 10.1155/2023/6449392 PMC12017038 40303723

[163] Yamaji R Yamada S Le M Q Identification of PB2 mutations responsible for the efficient replication of H5N1 influenza viruses in human lung epithelial cells J Virol 2015 89 07 3947 3956 25609813 10.1128/JVI.03328-14 PMC4403392

[164] Ungchusak K Auewarakul P Dowell S F Probable person-to-person transmission of avian influenza A (H5N1) N Engl J Med 2005 352 04 333 340 15668219 10.1056/NEJMoa044021

[165] Wang H Feng Z Shu Y Probable limited person-to-person transmission of highly pathogenic avian influenza A (H5N1) virus in China Lancet 2008 371 9622 1427 1434 18400288 10.1016/S0140-6736(08)60493-6

[166] Lemon S M Mahmoud A A The threat of pandemic influenza: are we ready? Biosecur Bioterror 2005 3 01 70 73 15853457 10.1089/bsp.2005.3.70

[167] Duan C Li C Ren R Bai W Zhou L An overview of avian influenza surveillance strategies and modes Science in One Health 2023 2 100043 10.1016/j.soh.2023.100043 PMC11262264 39077039

[168] Simancas-Racines A Cadena-Ullauri S Guevara-Ramírez P Zambrano A K Simancas-Racines D Avian influenza: strategies to manage an outbreak Pathogens 2023 12 04 610 37111496 10.3390/pathogens12040610 PMC10145843

[169] Martin V Forman A Lubroth J Preparing for Highly Pathogenic Avian InfluenzaQuébec City: Food and Agriculture Organization of the United Nations;2006

[170] Jonas O Warford L Global program for Avian Influenza control and human pandemic preparedness and response: project accomplishments. Health, Nutrition and Population (HNP) Discussion Paper Series;Washington, DC: The International Bank for Reconstruction and Development/The World Bank;2014(94043)

[171] Hay A J McCauley J W The WHO global influenza surveillance and response system (GISRS)-a future perspective Influenza Other Respir Viruses 2018 12 05 551 557 29722140 10.1111/irv.12565 PMC6086842

[172] Hill E M House T Dhingra M S The impact of surveillance and control on highly pathogenic avian influenza outbreaks in poultry in Dhaka division, Bangladesh PLOS Comput Biol 2018 14 09 e1006439 30212472 10.1371/journal.pcbi.1006439 PMC6155559

[173] Kingstad-Bakke B A Chandrasekar S S Phanse Y Effective mosaic-based nanovaccines against avian influenza in poultry Vaccine 2019 37 35 5051 5058 31300285 10.1016/j.vaccine.2019.06.077

[174] Zhang F Wang Y Shang X Production of high-titer infectious influenza pseudotyped particles with envelope glycoproteins from highly pathogenic h5n1 and avian h7n9 viruses J Vis Exp 2020 155 e60663 10.3791/60663 32009653

[175] Wu X Xiao L Li L Research progress on human infection with avian influenza H7N9 Front Med 2020 14 01 8 20 31989396 10.1007/s11684-020-0739-z PMC7101792

[176] Huang S W Tai C H Hsu Y M Assessing the application of a pseudovirus system for emerging SARS-CoV-2 and re-emerging avian influenza virus H5 subtypes in vaccine development Biomed J 2020 43 04 375 387 32611537 10.1016/j.bj.2020.06.003 PMC7274974

